# Hydration effects on the efficacy of the Epidermal growth factor receptor kinase inhibitor afatinib

**DOI:** 10.1038/s41598-017-01491-z

**Published:** 2017-05-08

**Authors:** Srinivasaraghavan Kannan, Mohan R. Pradhan, Garima Tiwari, Wei-Chong Tan, Balram Chowbay, Eng Huat Tan, Daniel Shao-Weng Tan, Chandra Verma

**Affiliations:** 1 Bioinformatics Institute (A*STAR), 30 Biopolis Street, 07-01 matrix, Singapore, 138671 Singapore; 2 Division of Medical Oncology, National Cancer Centre Singapore, 11 Hospital Drive, Singapore, 169610 Singapore; 3 Clinical Pharmacology Laboratory, National Cancer Centre Singapore, 11 Hospital Drive, Singapore, 169610 Singapore; 4 Cancer Therapeutics Research Laboratory, National Cancer Centre Singapore, 11 Hospital Drive, Singapore, 169610 Singapore; 5 Cancer Stem Cell Biology, Genome Institute of Singapore, 60 Biopolis Street, 02-01, Singapore, 138672 Singapore; 6 School of Biological Sciences, Nanyang Technological University, 60 Nanyang Drive, Singapore, 637551 Singapore; 7 Department of Biological Sciences, National University of Singapore, 14 Science Drive 4, Singapore, 117543 Singapore

## Abstract

Small molecules targeting the *EGFR* tyrosine kinase domain have been used with some success at treating patients with non-small cell lung cancer driven by activating mutations in the kinase domain. The initial class of inhibitors displaced ATP noncovalently but were rendered ineffective due to the development of resistance mutations in the kinase domain. These were overcome by the development of covalent inhibitors such as afatinib which also bind in the ATP pocket. However pooled analysis of two recent clinical trials LUX-3 and LUX-6 demonstrated an unprecedented overall survival benefit of afatinib over chemotherapy for the *EGFR* ^*19del*^, but not the *EGFR* ^*L858R*^. In the current study we use modelling and simulations to show that structural constraints in *EGFR* ^*19del*^ deletion result in significantly attenuated flexibilities in the binding pocket resulting in strong hydrogen and halogen bonds with afatinib in the *EGFR* ^*19del*^; these constraints are modulated by buried water and result in the differential affinities of afatinib for the different mutants. SNP analysis of residues surrounding the buried water points to the likelihood of further differential effects of afatinib and provides a compelling case for investigating the effects of the SNPs towards further stratification of patients for ensuring the most effective use of afatinib.

## Introduction

Diverse activating mutations within the *EGFR* kinase domain (KD) give rise to Non-small cell lung cancer (NSCLC). Structurally, the KD exists either in an inactive state or in a functional active state that is primed to bind ATP and substrate proteins. Treatment of NSCLC includes the inhibition of the kinase by Tyrosine Kinase Inhibitors (TKIs) such as erlotinib and gefitinib which are small molecules that bind to the KD and either compete out the binding of ATP or bind to the inactive state of the kinase. They have shown superior progression free survival when compared to cytotoxic chemotherapy and are currently approved for first line treatment of advanced *EGFR* mutant NSCLC where the commonest subtypes include the mutant L858R (40%) or exon 19 deletions (40%)^1^. However, resistance develops in the form of point mutations in the KD such as T790M which reduce the ability of these first generation small molecular inhibitors to bind effectively. Several biochemical and kinetic studies^2–4^ have shown that the T790M, L858R and the T790M/L858R double mutants have increased sensitivity towards the natural substrate (ATP) as compared to WT, usually by preferential stabilization of the active state resulting in decreased binding of the *EGFR* inhibitors. The structure of the KD of *EGFR* ^*WT*^ [Figure S1] and of the mutants (T790M) show that the substitution of the longer Met sidechain in the place of the shorter Thr side chain at position 790, which lies in the active site, results in steric hindrance of these inhibitors^5^. However, examination of the crystal structures of the active form of the WT and the L858R mutant shown that the KD adopts very similar structures in the active state [Figure S2]. Hence, it is not clear how binding is reduced by substitution of the hydrophobic leucine with a larger, positively charged arginine in L858R, which lies in the N-terminal portion of the activation loop, a region not at the inhibitor/ATP binding site. Long MD simulations^6^ have suggested that the L858R mutation results in stabilization of the active conformation of the KD by ordering the αC-helix (located in the N lobe of the kinase), resulting in enhanced dimerization. Similarly, metadynamics MD simulations^7^ suggested that these mutations shift the conformational equilibrium towards the active state. They found that the L858R mutation results in additional electrostatic interactions between R858 and the negatively charged residues E758, E762 or D761 from the αC-helix, resulting in reduced flexibility and stabilization of the KD in its active state. Co-crystal structures^8^ of inhibitors complexed to the KD of *EGFR* paved the path for the rational design of several second and third generation drugs to deal with the resistance mutations^8^ including the covalent inhibitor afatinib for treating *EGFR* ^*L858R*^ and *EGFR* ^*19del*^. In stably transfected *EGFR* mutant isogenic cell line models, afatinib inhibited phosphorylation in *EGFR* ^*19del*^ models to a higher extent than in *EGFR* ^*L858R*^, a finding that was not observed with reversible *EGFR* TKI^9^. Recently, Yang and colleagues reported a pooled analysis of two phase III trials for lung cancer (LUX-3 and LUX-6) comparing afatinib against platinum-based chemotherapy^10^. After a median follow up of 41 months, afatinib showed significant overall survival benefit over chemotherapy against the *EGFR* ^*19del*^ but not against the *EGFR* ^*L858R*^ mutation. Furthermore, additional subgroup analyses suggest that the overall survival benefit was observed across all patient cohorts regardless of the proportion of crossover^11^. The reason for this observation remains uncertain, although this difference was not previously seen with 1^st^ generation *EGFR* TKIs. While atomistic models of the L858R mutant and interactions with inhibitors are available, no such detailed information on *EGFR* ^*19del*^ is available, although the inhibitors are expected to bind as they do to *EGFR* ^*WT*^; however, the structural perturbations resulting from the deletion of five amino acids are expected to be significant. In this current study we seek to understand the mechanisms underlying the observed clinical differences in the effects of afatinib by developing atomistic models of the interactions of afatinib with the KD of *EGFR* ^*WT*^, *EGFR* ^*L858R*^, *and EGFR* ^*19del*^, we also carry out comparative MD simulations of the *EGFR* KD complexed to various first generation inhibitors (gefitinib, erlotinib; Figure S3).

## Results and Discussion

### Structural Basis underlying activating L858R and 19del mutations

In the crystal structures of apo and erlotinib bound *EGFR* ^*WT*^ ^3^, the hydrophobic sidechain of L858, which is part of the A-loop, points towards a charged and polar region of the substrate binding cleft (Figure S2). Upon mutation, the L858R sidechain assumes a polar character, but the crystal structure of *EGFR* ^*L858R*^ complexed with gefitinib does not show any structural perturbations, suggesting that the larger positively charged arginine side chain is readily accommodated. In the *EGFR* ^*19del*^ mutant, 5 amino acids (_746_ELREA_750_) that are part of a loop connecting the strand β3 with the αC-helix are deleted. This is expected to result in structural alterations in the KD as this long and flexible loop is thought to modulate the position and orientation of the αC-helix, which in turn is critical for the catalytic activity of the kinase^12, 13^. However no major structural differences were observed in our structural models of *EGFR* ^*19del*^ in either the apo or the inhibitor/ATP-bound states, relative to the corresponding wild type conformations. It is clear that the static structures or the two mutants cannot conclusively provide a mechanistic basis for the differential binding, and hence we explore the dynamical consequences of these mutations through MD simulations.

### MD simulations of *EGFR*^*L858R*^ and *EGFR*^*19del*^

**Figure 1:**
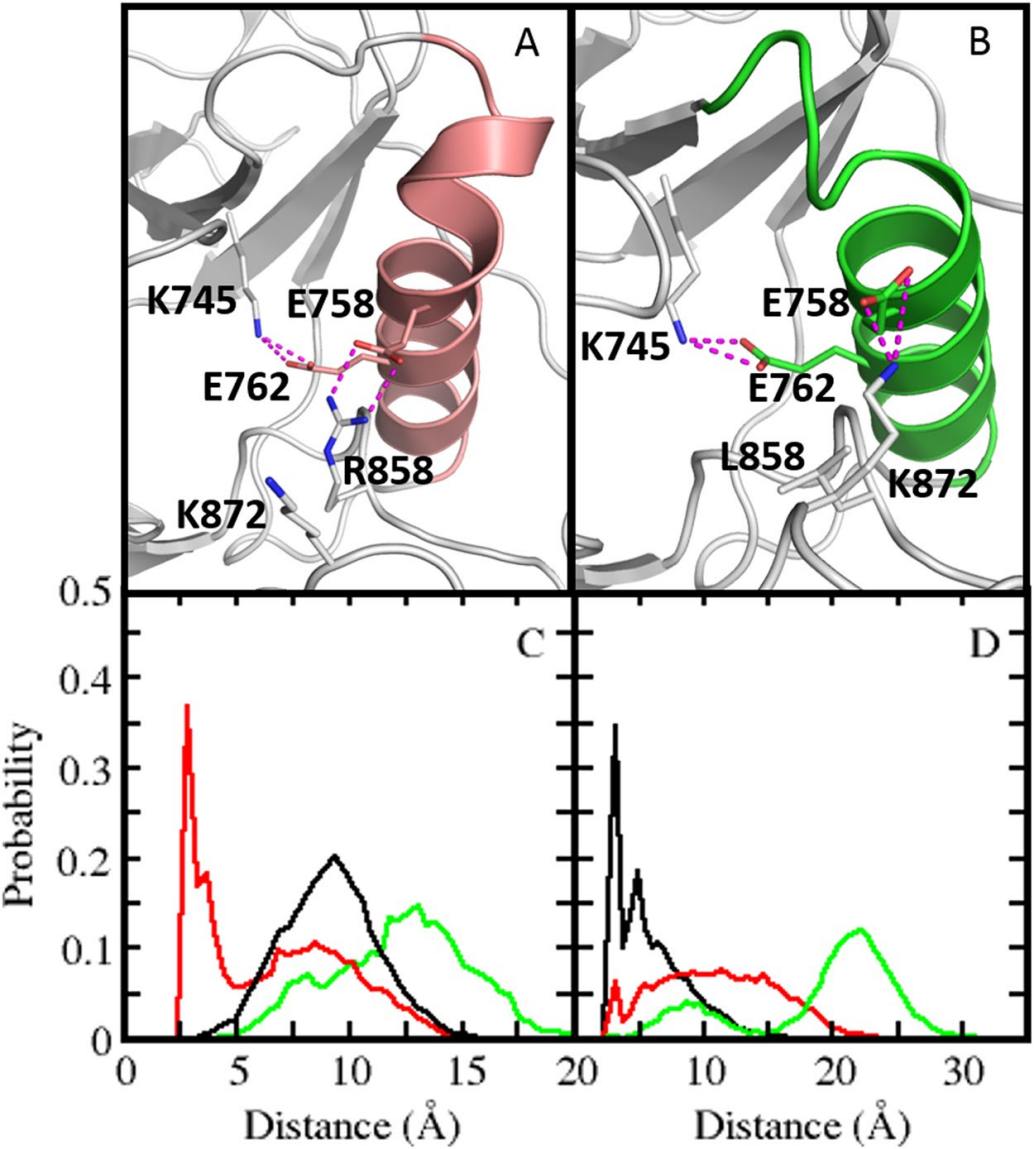
Mechanism of activation of the *EGFR* ^*L858R*^ and *EGFR* ^*19del*^. Snapshot of *EGFR* ^*L858R*^ (top left) and *EGFR* ^*19del*^ (top right highlighting the catalytically important salt bridge/interactions between K745 and E762 and a new interaction observed between R858 and E758 during the MD simulations. Probability distributions of distances (**A**) R858-E758 (**B**) K872-E758 sampled during MD simulations of *EGFR* ^WT^, *EGFR* ^*L858R*^ and *EGFR* ^*19del*^.

The *EGFR* ^*WT*^ structures were quite stable in both apo and inhibitor/ATP bound states, and the interactions observed in the co-crystal structures of wild type enzyme were well preserved during the MD simulations. In the wild type structures of *EGFR*, L858 stays buried. However, in the *EGFR* ^*L858R*^ simulations, the R858 flips out and interacts with the negatively charged residue E758 from the αC-helix (Figs 1 and S2; see Supplementary Movie), as has also been shown by Sutto and Gervasio^7^.

**Figure 2:**
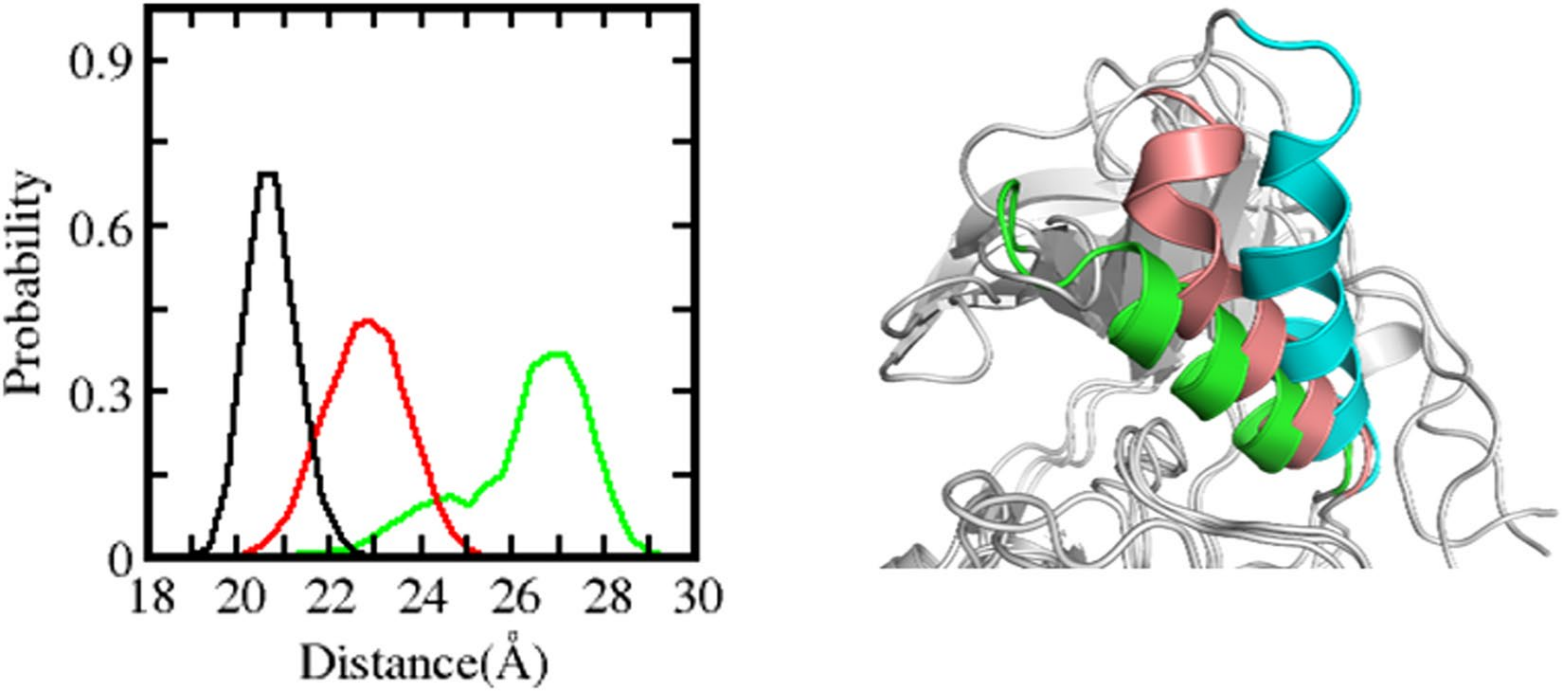
Distribution of ligand binding pocket size. Left: Distances between αC-helix and binding site of conformations sampled during the MD simulations of *EGFR* ^WT^ (green), *EGFR* ^L858R^ (red) and *EGFR* ^*19del*^ (black) in their apo states; Right: Snapshot of *EGFR* ^WT^ (cyan), *EGFR* ^*L858R*^ (brown) and *EGFR* ^*19del*^ (green) highlighting the movement of αC-helix during the MD simulations.

A salt bridge between R858 and E758 is well preserved throughout the simulation as the distance between the two side chains remains <3.5 Å (Fig. 1) in ~40% of the sampled conformations. However the corresponding residues in the *EGFR* ^*WT*^ and *EGFR* ^*19del*^ (both have Leu at position 858) are separated by ~12 Å and ~8 Å respectively (Fig. 1); this is not surprising as the L858 side chain cannot engage in electrostatic interactions with the αC-helix, while R858 can. We also observed interactions between R858 and E762 that were reported by Sutto and Gervasio^7^, however these were relatively short lived, existing in only ~8% of the sampled conformations. In summary *EGFR* ^*L858R*^ has additional electrostatic interactions between the A-loop R858 and the αC-helix. This results in the αC-helix moving towards the ATP-ligand binding cleft, stabilizing in the αC-in conformation and thus the active state of the KD. These motions are associated with a compaction of the ATP binding site, as seen by a reduction in the distance between the αC-helix and the hinge region by ~4 Å as compared to the *EGFR* ^*WT*^ (Fig. 2).

In the *EGFR* ^*19del*^ model, 5 amino acids (_746_ELREA_750_) that connect the αC-helix with the strand β3 are deleted. This region is thought to act as a flexible linker between αC and β3 and regulate the movement of αC between its αC-in and αC-out conformations. During the simulation of *EGFR* ^*19del*^ in the active state of the KD, unfolding at the N-terminus of the αC was observed, with the unfolded region now acting as the new linker between αC and β3. Despite this, the catalytically important K745-E762 salt bridge is well preserved throughout the simulation [Figure S4]. Shortening of the β3-αC loop reduces the intrinsic flexibility of the αC-helix, causing the αC-helix to be pulled in towards the ligand binding cleft [Fig. 2]. This results in a more compact closed pocket, with the distance between the αC helix and the hinge region of the kinase decreasing from ~27 Å in the case of *EGFR* ^*WT*^ to ~20.5 Å in *EGFR* ^*19del*^ [Fig. 2]. The inward movement of αC-helix enables additional interactions that likely further add to the stability of αC in its αC-in conformation. The K872 from the A-loop interacts with the E758 from the αC-helix, as the distance between the two side chains is <3.5 Å in ~40% of the sampled conformations [Fig. 1]. In contrast, this interaction was not observed during the *EGFR* ^*WT*^ simulation (distance between the two sidechains >~20 Å) [Fig. 1] as the αC-helix is not optimally positioned to interact with K872 from the A-loop. In the case of *EGFR* ^*L858R*^, the interactions between E758 from αC-helix and R858 from the A-loop [Fig. 1] appear to restrict interactions between K872 and E758; this interaction is seen in only <10% of the sampled conformations as the distance between the two side chains ranges from ~3–15 Å. The shortening of the loop will result in a significant barrier to adopt the inactive form, as the active to inactive transition requires the αC-helix to rotate and move away from its inward state to adopt the αC-out conformation. The *EGFR* ^*19del*^ mutation clearly stabilizes the active form, by reducing the flexibility of the αC-helix stabilized in the αC-in conformation, through additional interactions. In summary, both the mutations (*L858R* and *19del*) favour the active state of the kinase by stabilising the αC-helix in its αC-in conformation, by controlling the conformational flexibility of the αC – helix and/or through additional electrostatic interactions. This prepositioning of the αC-helix in its αC-in conformation is the likely driver for the observed enhanced dimerization that is associated with activation^14^.

**Figure 3:**
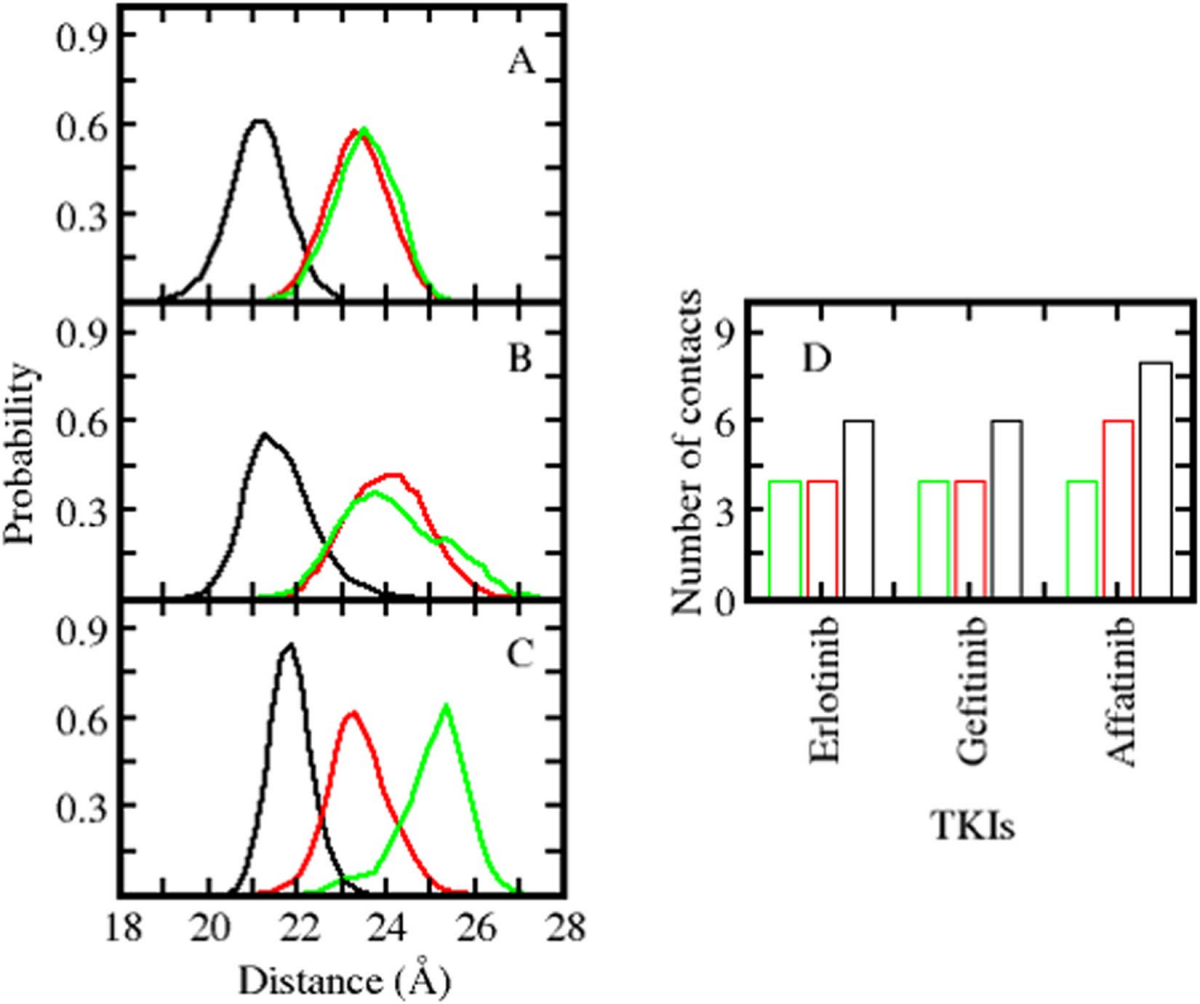
Distribution of ligand binding pocket size. Distances between αC-helix and binding site of conformations sampled during the MD simulations of *EGFR* ^WT^ (green), *EGFR* ^L858R^ (red) and *EGFR* ^*19del*^ (black) complexed to (**A**) afatinib (**B**) gefitinib (**C**) erlotinib state. (**D**) Average number of contacts that various inhibitors make with the alpha C-helix of *EGFR* ^WT^ (green), *EGFR* ^*L858R*^ (red) and *EGFR* ^*19del*^ (black).

The compaction of the ATP binding site in the active states of the KD of the *EGFR* mutants (Fig. 2 shows that compaction decreases as *EGFR* ^*19del*^ > *EGFR* ^*L858R*^ > *EGFR* ^*WT*^) results in tighter packing of the TKIs (Fig. 3A,B,C) with the density of inter-atomic contacts between the TKIs and the KD decreasing as: *EGFR* ^*19del*^ > *EGFR* ^*L858R*^ > *EGFR* ^*WT*^ (Fig. 3D); this order nicely mirrors the trend in the experimentally determined affinities of these inhibitors^2–5, 8^. To further quantify the differential binding of the TKIs with various mutants of EGFR, we calculated their binding free energies using the MMPBSA approach^15, 16^. Of course afatinib itself is covalently bonded to EGFR and hence one cannot use these classical methods to compute the binding energetics (these can be carried out using quantum mechanical methods^17^, but this is outside the scope of the current study). Therefore we calculated the binding free energies of erlotinib and gefitinib to the wildtype and mutant (L858R/19del) EGFR to explore whether the models we construct are in accord with experiments. It is clear that the calculations mirror the experimental trends in the binding affinities (experimental IC_50_, Figure S5). The differences between the experimental affinities for the wild type EGFR at first glance are much larger than the calculated ones; however, upon careful examination of the experimental data available in the literature [2–5, 8] we find that the spread in these values for the wild type EGFR is quite large and this is now shown through the error bars.

### Water molecules contribute to the binding patterns

The direct contacts between the inhibitors and the KD are often complemented by interactions that are mediated by buried water molecules, further modulating recognition and affinities^18^. Fernandez^19^ reported differences in the patterns of (de)hydration of the binding site of the inhibitor gleevec in two closely related kinases (Bcr-Abl, c-Kit). This was exploited in the modification of gleevec with a single methyl group to displace loosely bound waters in c-Kit, resulting in enhanced potency and selectivity towards c-Kit (while suppressing Bcr-Abl inhibition). Barillari *et al*.^20^ analysed the hydration of the ATP binding site across several kinases and suggested that the differences could be exploited to improve potency and selectivity of kinase inhibitors. A computational method called Watermap^21^ later rationalized the differing affinities of a range of kinase inhibitors based on differences in ATP site hydration. In a more recent exhaustive study, Nicholas and Steven^22^ reported that water mediated hydrogen bonds confer selectivity to bosutinib, another Bcr-Abl inhibitor used in the clinic, through a nitrile moiety. They found a remarkable correlation between an inhibitor’s access to structured water molecules and its ability to distinguish between receptor subtypes. It was shown recently that interfacial water molecules can have a substantial effect on the thermodynamics of ligand binding^23^. Sorbinil is involved in water mediated interaction with aldose reductase and this water mediated hydrogen bond contributes ~−5.1 kJ/mol to its binding. Interfacial waters have also been previously reported to facilitate non-specific binding as seen in the transport of a range of peptides up to 5 residues in length by the oligopeptide binding protein OppA^24^. In general there is growing realization of the importance of waters in modulating protein-protein/peptide/small molecule interactions^25, 26^.

In addition to the role of water, recently, the role of halogen atoms (F, C, Br and I; several drug candidates are halogenated including 20% of ligands in the pdb^27^) in contributing to affinity and selectivity through “halogen bonds”^28–30^ is being recognized, both through direct contacts with the KD and through buried water^31^. Since both gefitinib and afatinib are halogenated, we carried out detailed analyses of water mediated hydrogen bonds and halogen bond interactions.

**Figure 4:**
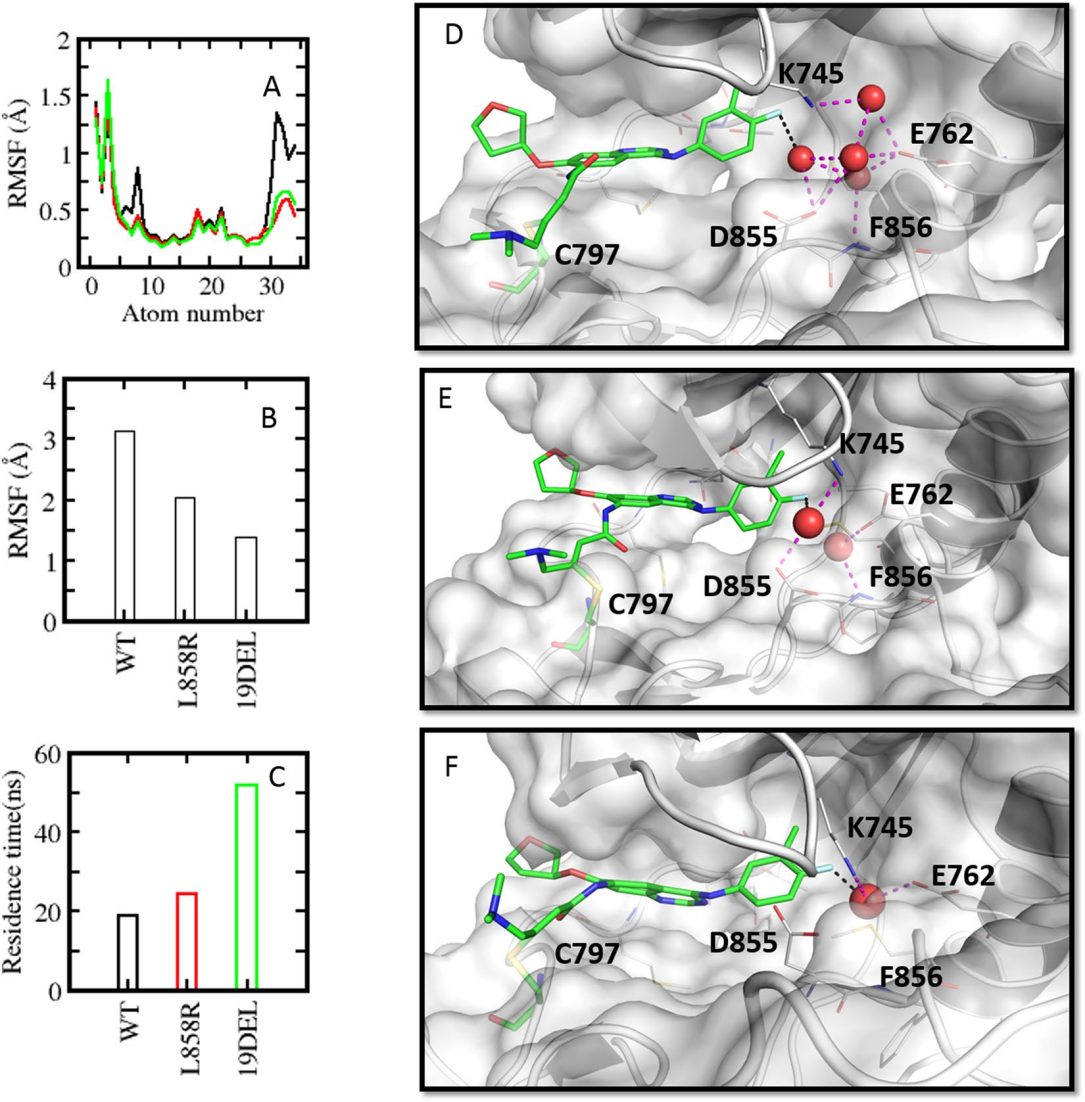
(**A**) Atomic root mean square fluctuations (rmsf) of afatinib bound to *EGFR* ^WT^ (black) *EGFR* ^L858R^ (red) *EGFR* ^*19del*^ (green) sampled during the MD simulations. (**B**) rmsf of αC-helix sampled during the MD simulations of *EGFR* ^WT^, *EGFR* ^L858R^, *EGFR* ^*19del*^ complexed with afatinib. (**C**) residence times of water molecules observed during the MD simulations of *EGFR* ^WT^ (black) *EGFR* ^L858R^ (red) *EGFR* ^*19del*^(green) complexed to afatinib. Water molecules observed between the inhibitors and αC helix of *EGFR* ^WT^ (**D**), *EGFR* ^*L858R*^ (**E**), *EGFR* ^*19del*^ (**F**). Hydrogen bond (magenta) and halogen bond (black) interactions are shown as dotted lines.

Gefitinib was stabilized in *EGFR* ^*19del*^ and *EGFR* ^*L858R*^ by 2 and 3 water molecules respectively (residence times of ~28 ns and ~24 ns), while erlotinib was only stabilized weakly (water molecules with low residence times of 8 ns and 12 ns in *EGFR* ^*19del*^ and *EGFR* ^*L858R*^) [Figure S6]. In contrast, when bound to afatinib, both mutants appear to undergo dehydration, leaving one strongly bound water molecule in *EGFR* ^*19del*^ with residence time of ~50 ns and 2 loosely bound water molecules in *EGFR* ^*L858R*^ with residence times of ~25 ns [Fig. 4].

The reason for these differences in hydration appears to originate in the differences in flexibilities of both the KD and the inhibitors, as well as in specific water-KD and water-ligand interactions. Both mutants stabilize the active state, restricting the flexibility of the αC-helix and compacting the inhibitor binding site as compared to *EGFR* ^*WT*^ (this is more pronounced in *EGFR* ^*19del*^ than in *EGFR* ^*L858R*^). Recently, a similar stabilization of the active state of the equivalent del mutant has been shown for the first time in a crystal structure of BRAF [5HID]^32^. In the case of afatinib, the average fluctuations of αC -helix are 3.2, 2.2 and 1.3 Å respectively for the *EGFR* ^*WT*^, *EGFR* ^*L858R*^ and *EGFR* ^*19del*^ mutants [Fig. 4B]. This reduced flexibility of the αC-helix is coupled to a reduction in the flexibility of afatinib (the region of afatinib near the helix undergoes a reduction in  average fluctuation from ~1.2 to ~0.5 Å between the wild type and the mutants) [Fig. 4A]. The reduced flexibility of αC-helix and afatinib is associated with the increased residence time observed for water molecules that stabilize the binding of afatinib. In the case of the *EGFR* ^*19del*^ – afatinib complex, one tightly bound water molecule bridges the αC-helix with afatinib through the sidechain of E628 (αC-helix) and the fluorine atom on afatinib (this halogen bond decreases in length from 3.9 Å in the wild type to 3.2 Å in *EGFR* ^*L858R*^ and 2.7 Å in *EGFR* ^*19del*^). Reduced flexibility of αC-helix results in trapping of water molecules in the space between the helix and afatinib which in turn may well be the cause of the observed reduction in flexibility of afatinib and hence it’s tight binding. As the fluctuation of the αC-helix increases (relatively) in the case of *EGFR* ^*L858R*^ and *EGFR* ^*WT*^, the increased space is filled by more water molecules; the resulting absence of strong stabilising interactions results in the water molecules gaining greater mobility.

Gefitinib also has a halogen atom (F) similar to afatinib, and this F is also involved in water mediated h-bonds and halogen-bond interactions with the αC-helix of the KD. However a tightly bound water with higher residence time was not observed even when complexed with the more compact *EGFR* ^*19del*^, as gefitinib exhibits increased flexibility compared to afatinib. The increased flexibility observed for gefitinib in general arises from the noncovalent nature of its interaction with the KD; in contrast, afatinib makes a covalent bond with Cys797 in the KD which restricts its mobility. Due to the increased flexibility of gefitinib, the water mediated halogen bond interaction is not stable resulting in a loosely bound trapped water molecule. In contrast, erlotinib lacks a halogen atom, and the reduced interaction results in higher flexibility and only one loosely bound water molecule with residence times of ~8 to 14 ns in its complexes with *EGFR* ^*WT*^, *EGFR* ^*L858R*^ and *EGFR* ^*19del*^ [Figure S6].

In summary, the tightly bound water molecule in the case of afatinib results from trapping arising from two constraints - the covalent bond and the reduced ATP pocket coupled to constrained mobility in *EGFR* ^*19del*^ (the water is trapped in a largely hydrophobic region), resulting in strong water mediated hydrogen bond and halogen bond interactions. The water mediated hydrogen bond between the catalytic K745 and E762 from the αC-helix has been widely observed as stabilizing kinase-ATP/inhibitor complexes^12, 13, 19–22^. Several studies have highlighted the importance of water mediated protein – ligand interactions in improving potency and selectivity of inhibitors. In this regard, the contributions from well-structured/ordered water molecules have provided significant insights into contributions for improving the binding affinity in several examples^19–26^. In addition to this interactions, the water mediated halogen bond interaction between the KD and inhibitor that is observed in the *EGFR* ^*19del*^-afatinib simulations clearly suggests a significant component in enhancing the binding of Afatinib to *EGFR* ^*19del*^ over *EGFR* ^*WT*^ and *EGFR* ^*L858R*^. The waters in the case of the other two drugs are loosely bound because both drugs make noncovalent interactions and hence trap waters under only one constraint - that of the compaction arising from the exon 19 deletion. Taken together these structural interactions may account for the increased activity for afatinib in exon 19 deletions, resulting in subsequent improved overall survival, as observed in the pooled analysis of LUX3 and LUX6. These models are consistent with the emerging preclinical and clinical data with regards to preferential activity of afatinib in *EGFR* ^*19del*^, and highlight the need to prospectively validate these prediction models in the clinic.

We finally wondered whether we could use our models to speculate on further stratification of patients through examination of SNP data. If we examine the distribution of SNPs^33^ in the region within 5 Å of the water molecule in the del19 mutant, we find that several residues [Figure S7] can vary in the population. Simple modelling of the SNPs M766T and M766F by mutating the sidechains without accounting for any other perturbation shows [Figure S7] that the changes in the number of atoms in the sidechain and hence the shape of the hydration cavity has the potential to change interactions with afatinib either directly or through an altered number of water molecules, thus affecting the affinity for afatinib. It is already known^34^ that mutations at M766, affect the binding of erlotinib. We are currently carrying out an exhaustive simulation based study exploring these effects. These observations provide a compelling case for experimental tests of the effects of the SNPs on the binding of afatinib. If validated, this points to the possibility of further sub-stratification of patients amongst the cohort carrying the del19 mutations for afatinib treatment. In addition, it opens up a new approach to patient stratification guided by molecular modelling in this era of personalized medicine.

## Methods

In order to understand the structural basis of activation of the activating mutations and differential binding of TKIs we have used atomistic molecular dynamics (MD) simulations. The KD structures were taken either from the experimental database of structures or else modelled based on standard comparative modelling methods^35^. Since these mutations are known to be activating and the inhibitors/ATP considered here are known to bind to the active forms of the KDs, only the active form of *EGFR* ^*WT*^, *EGFR* ^*L858R*^, *and EGFR* ^*19del*^ are considered in this study.

Experimental structures of *EGFR* ^*WT*^, in its apo form and bound with various inhibitors are available and are used here. In the case of *EGFR* ^*L858R*^, only structures of *EGFR* ^*L858R*^ complexed with TKIs available and the apo active form was modelled. No crystal structures of *EGFR* ^*19del*^ is available, and was therefore modelled using computational modelling approaches. Details of the structures and templates used in this study are listed in Table S1. Models of *EGFR* ^*WT/L858R/19del*^ - inhibitor complexes were also generated using the available co-crystal structures. All these modelled systems were then subject to atomistic MD simulations.

### MD Simulations

MD simulations were carried out with the *Sander* module of the program Amber11^36^. The partial charges and force field parameters for each inhibitor were generated using the *Antechamber* module in Amber. All atom versions of the Amber 99SB force field (ff03)^37^ and the general Amber force field (GAFF)^38^ were used for the protein and the inhibitors respectively. In our simulations the inhibitor afatinib is covalently linked to Cys797 from *EGFR*. Therefore parameters for afatinib covalently bonded with Cys was derived using antechamber module of Amber 11. The *Xleap* module was used to prepare the system for the MD simulations. All the simulation systems were neutralized with appropriate numbers of counter ions. Each neutralized system was solvated in an octahedral box with TIP3P^39^ water molecules, leaving at least 10 Å between the solute atoms and the borders of the box. All MD simulations were carried out in explicit solvent at 300 K. During the simulations, the long-range electrostatic interactions were treated with the particle mesh Ewald^40^ method using a real space cut off distance of 9 Å. The Settle^41^ algorithm was used to constrain bond vibrations involving hydrogen atoms, which allowed a time step of 2 fs during the simulations.

Solvent molecules and counter ions were initially relaxed using energy minimization with restraints on the protein and inhibitor atoms. This was followed by unrestrained energy minimization to remove any steric clashes. Subsequently the system was gradually heated from 0 to 300 K using MD simulations with positional restraints (force constant: 50 kcal mol^−1^ Å^−2^) on protein and inhibitors over a period of 0.25 ns allowing water molecules and ions to move freely. During an additional 0.25 ns, the positional restraints were gradually reduced followed by a 2 ns unrestrained MD simulation to equilibrate all the atoms. All the simulations were carried out in triplicates (three independent MD simulations initiated with different initial velocities) for 100 ns with conformations saved every 10 ps. Molecular Mechanics Poisson Boltzmann Surface Area (MMPBSA) method^15, 16^ was used for the calculation of binding free energies (more details in Supplementary Information).

### Analysis

Root mean square deviation of sampled conformations against the starting structure and average atomic fluctuations of all sampled conformations during MD simulations were calculated using ptraj module in Amber. The distance between R858-E758 and K872-E758 were calculated by measuring the distance between the side chain atoms of R858 and the side chain atoms of E758 and similarly the side chain atoms of K872 and the side chain atoms of E758. The ATP/TKIs binding pocket size was measured by calculating the distance between the centre of mass (COM) of residues from αC helix and COM of residues from the kinase hinge region. The number of kinase-inhibitor contacts were calculated by considering all heavy atom pairs of kinase-inhibitors that are within 6.5 Å. A hydrogen bond was considered when the donor-acceptor distance was less than 3.0 Å and the angle formed by donor-hydrogen-acceptor was >120°. To further understand the role of water mediated kinase-inhibitor interactions, analysis was carried out with water molecules that are within 3.5 Å of inhibitor and kinase residues. The water residence time at a particular site was computed as a summation of times over the trajectory during which the corresponding site has a water molecule occupying it. If a water molecule were to leave a site and another were to exchange with it, the calculation is agnostic to the identity of the water and only accounts for the hydration of the site. Simulation trajectories were visualized using VMD^42^ and figures were generated using PyMOL^43^.

## Electronic supplementary material

Supplementary movie

Supplementary information

## References

[1] Tan DS (2016). The International Association for the Study of Lung Cancer consensus statement on optimizing management of EGFR mutation-positive non-small cell lung cancer: status in 2016. J. Thorac. Oncol..

[2] Yun CH (2008). The T790M mutation in EGFR kinase causes drug resistance by increasing the affinity for ATP. Proc. Natl. Acad. Sci. USA.

[3] Yun CH (2007). Structures of lung cancer-derived EGFR mutants and inhibitor complexes: Mechanism of activation and insights into differential inhibitor sensitivity. Cancer Cell..

[4] Carey KD (2006). Kinetic analysis of epidermal growth factor receptor somatic mutant proteins shows increased sensitivity to the epidermal growth factor receptor tyrosine kinase inhibitor Erlotinib. Cancer Res..

[5] Kobayashi S (2005). EGFR mutation and resistance of non-small-cell lung cancer to Gefitinib. N. Engl. J. Med..

[6] Shan Y (2012). Oncogenic mutations counteract intrinsic disorder in the EGFR kinase and promote receptor dimerization. Cell..

[7] Sutto L, Gervasio FL (2013). Effects of oncogenic mutations on the conformational free-energy landscape of EGFR kinase. Proc. Natl. Acad. Sci. USA.

[8] Singh D, Attri BK, Gill RK, Bariwal J (2016). Review on EGFR inhibitors: critical updates. Mini. Rev. Med. Chem.

[9] Banno E (2015). Afatinib is especially effective against non-small cell lung cancer carrying an EGFR exon 19 deletion. Anticancer Res..

[10] Yang JC (2015). Afatinib versus cisplatin-based chemotherapy for EGFR mutation-positive lung adenocarcinoma (LUX-Lung 3 and LUX-Lung 6): analysis of overall survival data from two randomised, phase 3 trials. The Lancet Oncology.

[11] Yi-Long, W. *et al*. Impact of dose adjustment on Afatinib safety and efficacy in epidermal growth factor receptor mutation-positive nonsmall cell lung cancer: post-hoc analyses of LUX-Lung 3/LUX-Lung 6. *Asia Pacific Lung Cancer Conference* (2016).

[12] Johnson LN, Noble ME, Owen D (1996). Active and inactive protein kinases: structural basis for regulation. Cell..

[13] Huse M, Kuriyan J (2002). The conformational plasticity of protein kinases”. Cell..

[14] Valley CC (2015). Enhanced dimerization drives ligand-independent activity of mutant epidermal growth factor receptor in lung cancer. Mol Biol Cell.

[15] Hou T, Wang J, Li Y, Wang W (2011). Assessing the performance of the MM/PBSA and MM/GBSA methods. 1. The accuracy of binding free energy calculations based on molecular dynamics simulations. J. Chem. Inf. Model..

[16] Genheden S, Ryde U (2015). The MM/PBSA and MM/GBSA methods to estimate ligand-binding affinities. Expert Opin. Drug Discov.

[17] Bruice TC (2006). Computational approaches: reaction trajectories, structures, and atomic motions. Enzyme reactions and proficiency. Chem. Rev.

[18] Hu J (2015). Kinase regulation by hydrophobic spine assembly in cancer. Mol. Cell. Biol..

[19] Fernández A (2007). An anticancer C-Kit kinase inhibitor is reengineered to make it more active and less cardiotoxic. J. Clin. Invest..

[20] Barillari C (2011). Analysis of water patterns in protein kinase binding sites. Proteins..

[21] Robinson DD, Sherman W, Farid R (2010). Understanding kinase selectivity through energetic analysis of binding site waters. ChemMedChem..

[22] Levinson NM, Boxer SG (2014). A conserved water-mediated hydrogen bond network defines bosutinib’s kinase selectivity. Nat. Chem. Biol..

[23] Klebe G (2015). Applying thermodynamic profiling in lead finding and optimization. Nature Reviews Drug Discovery.

[24] Tame JR, Sleigh SH, Wilkinson AJ, Ladbury JE (1996). The role of water in sequence-independent ligand binding by an oligopeptide transporter protein. Nature Structural Biology.

[25] Maestre-Reyna M, Diderrich R, Veelders MS, Eulenburg G, Kalugin V, Brückner S, Keller P, Rupp S, Mösch H-U, Essen L-O (2012). Structural basis for promiscuity and specificity during Candida glabrata invasion of host epithelia. Proc Natl Acad Sci USA.

[26] Mueller TD, Nickel J (2012). Promiscuity and specificity in BMP receptor activation. FEBS Lett.

[27] Poznański J, Poznańska A, Shugar D (2014). A Protein Data Bank Survey Reveals Shortening of Intermolecular Hydrogen Bonds in Ligand-Protein Complexes When a Halogenated Ligand Is an H-Bond Donor. PLoS ONE..

[28] Adasme-Carreño F, Muñoz-Gutierrez C, Alzate-Morales JH (2016). Halogen bonding in drug-like molecules: A computational and systematic study of the substituent effect. RSC Advances.

[29] Lu Y (2012). Halogen bonding for rational drug design and new drug discovery. Expert Opinion on Drug Discovery.

[30] Sirimulla S, Bailey JB, Vegesna R, Narayan M (2013). Halogen interactions in protein-ligand complexes: implications of halogen bonding for rational drug design. J Chem Inf Model.

[31] Poznanski J, Shugar D (2013). Halogen bonding at the ATP binding site of protein kinases: Preferred geometry and topology of ligand binding. Biochimica et Biophysica Acta.

[32] Foster SA (2016). Activation Mechanism of Oncogenic Deletion Mutations in BRAF, EGFR, and HER2. Cancer Cell..

[33] Forbes SA (2015). COSMIC: exploring the world’s knowledge of somatic mutations in human cancer. Nucleic Acids Res.

[34] Vizienyte E, Ward R, Garner A (2008). Comparison of the EGFR resistance mutation profiles generated by EGFR-targeted tyrosine kinase inhibitors and the impact of drug combinations. Biochem J..

[35] Sali A, Blundell TL (1993). Comparative protein modelling by satisfaction of spatial restraints. J. Mol. Biol..

[36] Case, D. *et al*. Amber 11. University of California, San Francisco (2011).

[37] Ponder JW, Case DA (2003). Case. Force fields for protein simulations. Adv. Prot. Chem.

[38] Wang J, Wolf RM, Caldwell JW, Kollman PA, Case DA (2004). Development and testing of a general amber force field. J. Comput. Chem..

[39] Jorgensen WL, Chandrasekhar J, Madura JD, Impey RW, Klein ML (1983). Comparison of simple potential functions for simulating liquid water. J. Chem. Phys..

[40] Darden T, York D, Pedersen L (1993). Particle mesh Ewald: An N_log(N) method for Ewald sums in large systems. J. Chem. Phys..

[41] Miyamoto S, Kollman PA (1992). Settle: An analytical version of the SHAKE and RATTLE algorithm for rigid water models. J. Comput. Chem..

[42] Humphrey W, Dalke A, Schulten K (1996). VMD—visual molecular dynamics. J. Mol. Graph..

[43] De Lano, W. The PyMOL molecular graphics system. San Carlos CA, USA: *De Lano Scientific* (2002).

