# Supplementary material for: Hydration effects on the efficacy of the Epidermal growth factor receptor kinase inhibitor afatinib
Source: Sci Rep. 2017 May 8;7:1540. doi: 10.1038/s41598-017-01491-z (PMC5431542; doi:10.1038/s41598-017-01491-z)
Supplement: Supplementary file 2 — Supplementary information [file 41598_2017_1491_MOESM2_ESM.doc]

**Supplementary information**

**Hydration effects on the efficacy of the anticancer drug afatinib**

Srinivasaraghavan Kannan^1*^, Mohan R. Pradhan^1^, Garima Tiwari^1^, Wei-Chong Tan^2^, Balram Chowbay^3^, Eng Huat Tan^2^, Daniel Shao-Weng Tan^2,4,5*^, Chandra Verma^1,6,7*^

**Table S1:** Details of systems studied here

| Substrate/Inhibitor | | Wildtype (WT) | L858R | Exon 19 deletion (_746_ELREA_750_) |
| --- | --- | --- | --- | --- |
| AMP-PNP | | 2ITX^a^ | 2ITV^a^ | Model |
| Gefitinib | | 2ITY^a^ | 2ITZ^a^ | Model |
| Erlotinib | | 1M17^b^ | Model | Model |
| Afatinib | | 4G5J^c^ | Model | Model |
| Apo | Active | 2ITX^a,d^ | 2ITV^a,d^ | Model |
|  | Inactive | 2GS7^e^ | Model | Model |

1. Yun CH et al. Structures of Lung Cancer-Derived *EGFR* Mutants and Inhibitor Complexes: Mechanism of Activation and Insights into Differential Inhibitor Sensitivity. *Cancer Cell.* **11(3),** 217-227 (2007).
2. Stamos J, Sliwkowski MX, Eigenbrot C. Structure of the epidermal growth factor receptor kinase domain alone and in complex with a 4-anilinoquinazoline inhibitor. *J Biol Chem*. **277,** 46265–46272 (2002).
3. Solca F et al. Target binding properties and cellular activity of Afatinib (BIBW 2992), an irreversible ErbB family blocker. *J Pharmacol Exp Ther*. **343(2),** 342-50 (2012).
4. Apo states were prepared by removing the bound ligand from the co-crystal structures.
5. Zhang X, Gureasko J, Shen K, Cole PA, Kuriyan J. An Allosteric Mechanism for Activation of the Kinase Domain of Epidermal Growth Factor Receptor. *Cell.* **125(6),** 1137-1149 (2006).

**
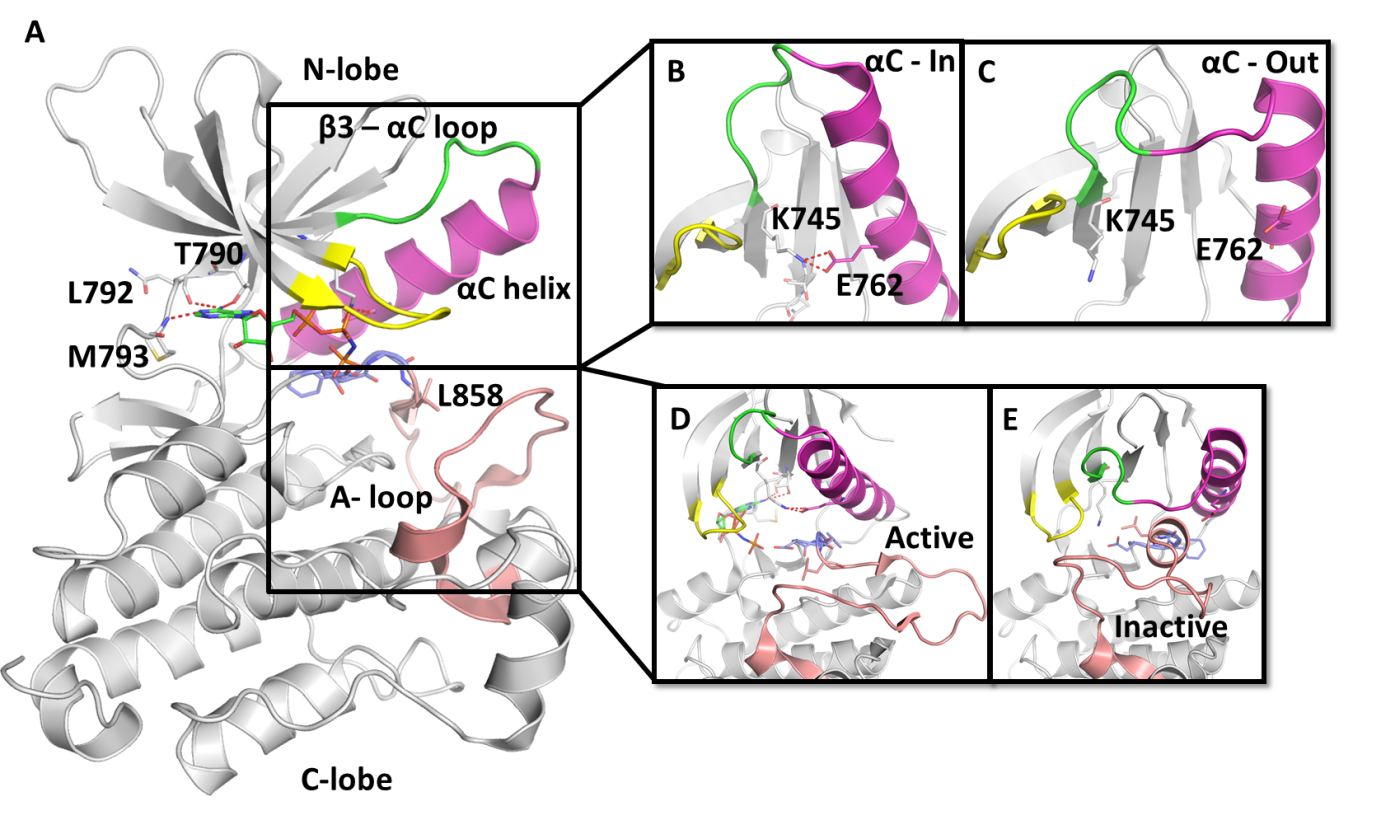
**

**FigureS1:** (A) overview of the structure of the *EGFR* kinase in its active form with the Glycine rich P-loop (yellow), αC – helix  (magenta), DFG motif (blue) and the activation loop (A-loop) (brown) highlighted. The structure of the wildtype kinase is shown in complex with the ATP analogue AMP-PNP and the interacting residues at the hinge region are highlighted in sticks. All the interactions are highlighted in dotted lines (magenta).  The location of L858 and the β3 – αC loop (corresponding to *EGFR* *^19del^*) (Green) are highlighted. Comparison of the conformation of the αC-helix in its active (αC – in) (B) and inactive (αC – out) (C) form with the Lys745 – Glu762 interaction highlighted. Comparison of the conformations of the activation loop in their active (D) and inactive form (E).

Multiple crystal structures of the KD of *EGFR* in its wild type and mutant states have shown that it exists in two conformations - termed active and inactive ^4, 5^ of which the active conformation uniquely binds ATP. The KD can be partitioned into two lobes: a smaller N-lobe and a larger C-lobe [Figure S1]. The N-lobe has a pre-dominantly antiparallel β-sheet structure (β1 to β5) and contains an important regulatory alpha helix, referred to as the αC-helix. It also contains the consensus glycine-rich (GSGAFG) ATP-phosphate binding loop. The C-lobe is pre-dominantly α-helical, but also contains four short conserved β strands that present most of the catalytic residues associated with the phosphoryl transfer from ATP to its protein or peptide substrates. The catalytic site/ATP-binding-site lies in a cleft between the two lobes, a pattern that is conserved amongst most known kinases ^4, 5^.  Three structural elements [Figure S1] within the kinase domain are hypothesized to control the active-inactive forms of the kinase: 1) αC-helix (residues 754-768); 2) activation loop or A-loop in the C-lobe (residues 858-884); 3) the DFG motif (residues Asp855, Phe856, Gly857) in the A-loop. In the active form, the αC-helix points in towards the ATP binding site and the DFG motif adopts a DFG-in conformation, which is characterized by Asp855 (D) facing into the ATP-binding pocket, Phe856 (F) pointing away from the ATP-binding pocket, and the A-loop forming an open extended structure (Figure S1D) that facilitates substrate binding. A salt bridge between a conserved Lysine (K745) from β3 strand and a glutamate (E762) from the αC-helix stabilizes the active state and is necessary for function. In the inactive form, the αC-helix is displaced from the αC-in to an αC-out conformation and the DFG motif attains the DFG-out conformation where Asp855 (D) faces away from the ATP-binding pocket, Phe856 (F) points into the ATP-binding pocket [Figure S1B, S1C] and the A-loop assumes a closed compact structure  [Figure S1E] that blocks the binding of substrate and the outward movement of αC-helix results in disruption of the salt bridge. Crystal structures of the inactive forms of *EGFR* show that it adopts a “Src-like” inactive conformation. This shows the A-loop with a short α-helix which engages in an autoinhibitory interaction with the αC-helix which also have moved “away”, while the DFG motif adopts a DFG-in conformation similar to that adopted in its active form. In summary, the correct positioning of the αC-helix and the A-loop together with interactions with a dimeric partner (can be the kinase domain of *EGFR* or HER2) are required for activation of the *EGFR* KD ^6^).  The oncogenic mutations in *EGFR* have been found to be largely clustered around the catalytic site/active site cleft ^7^.


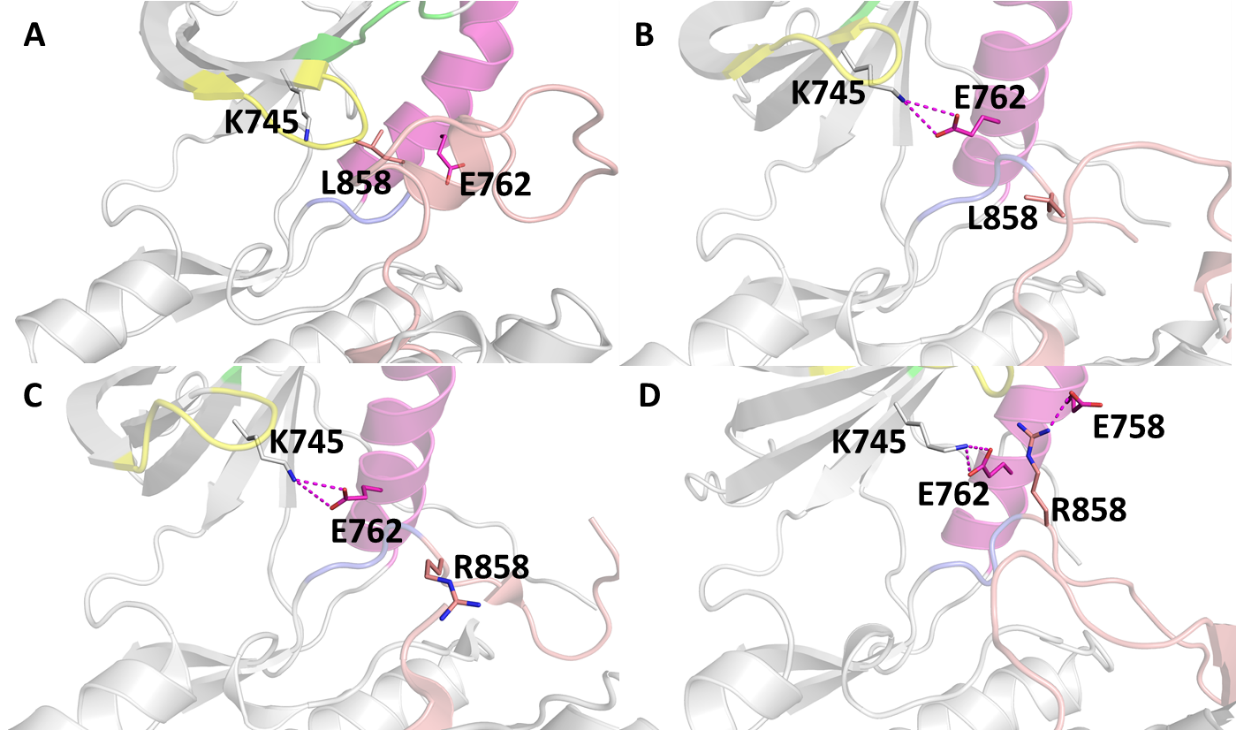


**Figure S2**: Mechanism of activation of the *EGFR* *^L858R^* mutant. Crystal structures of: (A) *EGFR ^WT^* in its inactive form; (B) *EGFR**^WT^* in its active form; (C) *EGFR* *^L858R^* in its active form; (D) MD snapshot of the *EGFR**^L858R^* mutant which adopts an active conformation. Catalytic important salt bridge/interactions between Lys745 and Glu762 and a new interaction observed between R858 and E758 during the MD simulations (D) are highlighted.


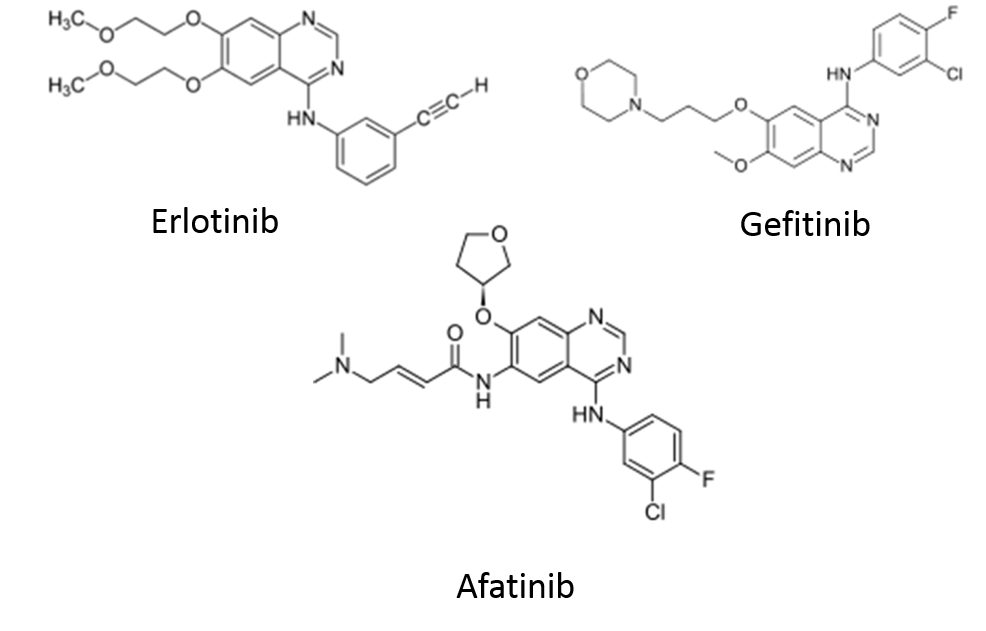


**Figure S3:** Structures of the drugs/inhibitors used in this study.


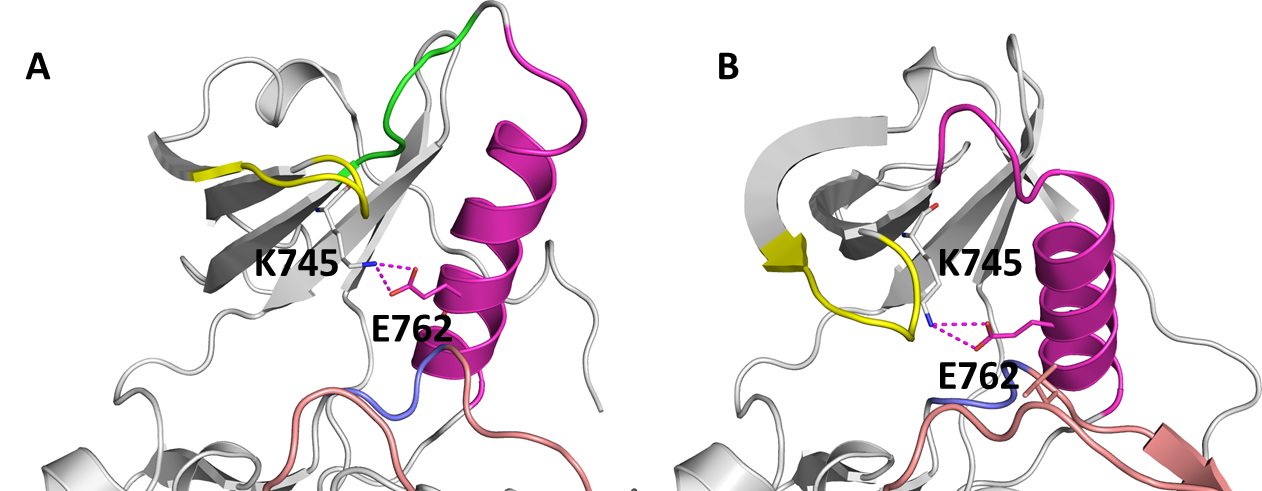


**Figure S4**: Mechanism of activation of the *EGFR^19del^* mutant (A) Crystal structure of *EGFR^WT^* in its active state (B) MD snapshot of *EGFR^19del^* mutant conformation in the active state with the catalytic important salt-bridge/interactions between Lys745 – Glu762 shown.


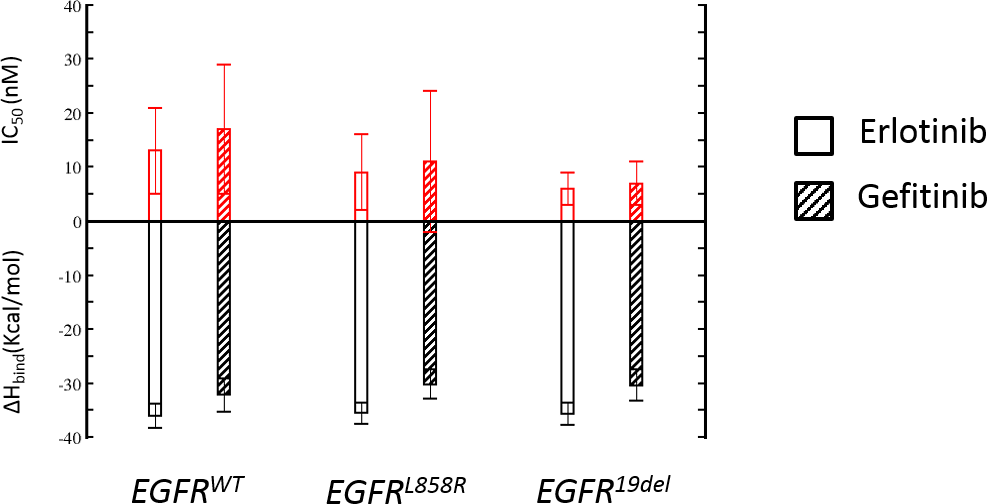


**Figure S5:** Comparison of the experimental IC_50_ (positive y-axis) of the binding of Erlotinib/Gefitinib to *EGFR* (WT, L858R, 19del) with the calculated binding energies (negative y-axis) of the corresponding complexes, using the MMPBSA method^15, 16^.

The MMPBSA (Molecular Mechanics Poisson–Boltzmann Surface Area) calculations^15, 16^ were carried out after removing the water molecules and the counterions. Binding free energies were calculated using the single trajectory method, based on the assumption that the bound and unbound conformations of the protein and inhibitor are quite similar. In this protocol, the isolated conformations of the inhibitor and the protein were extracted from the corresponding protein-inhibitor complex. For each conformation, the binding free energy (ΔG_bind_) of the inhibitor to the protein was calculated as follows:

Δ*G*_bind_ = *G*_complex_ − (*G*_receptor_ + *G*_ligand_) (1)

The binding free energy calculated here corresponds to only the enthalpic component of the total binding free energy, without any contributions from entropy. Entropy calculations are computationally intensive and do not converge easily and hence are ignored. Therefore the binding free energy is estimated as a sum of two terms:

Δ*H*_bind_ = Δ*G*_MM_ + Δ*G*_sol_ (2)

where ΔG_MM_ is the change in the molecular mechanics energy upon complexation in the gas phase, ΔG_sol_ is the change in the solvation free energy associated with ligand binding.

The molecular mechanics free energy (ΔG_MM_) is further split into Van der Waals (ΔG_vdw_) and electrostatic (ΔG_ele_) energies:

Δ*G*_MM_ = Δ*G*_ele_ + Δ*G*_vdw_ (3)

The solvation free energy ΔG_sol_ arises from polar (electrostatic) solvation free energy (ΔG_PB_) and nonpolar solvation free energy (ΔG_SA_) as in eq 4:

Δ*G*_sol_ = Δ*G*_PB_ + Δ*G*_SA_ (4)

ΔG_PB_ is computed by solving the linearized Poisson – Boltzmann (PB) equation using Parse radii and a solvent probe radius of 1.4 Ǻ. In our calculations, the dielectric constant was set to 1.0 for the interior of the solutes and 80.0 for the solvent. ΔG_SA_ was determined using a solvent accessible surface area (SASA)–dependent term as in eq 5:

Δ*G*_SA_ = *γ* × SASA + *β* (5)

where γ is the surface tension proportionality constant and was set to 0.00542 kcal/(mol·Å^−2^), and β is the offset value, which was 0.92 kcal/mol here. MMPBSA binding energies were calculated using the MMPBSA.py module in Amber 11.


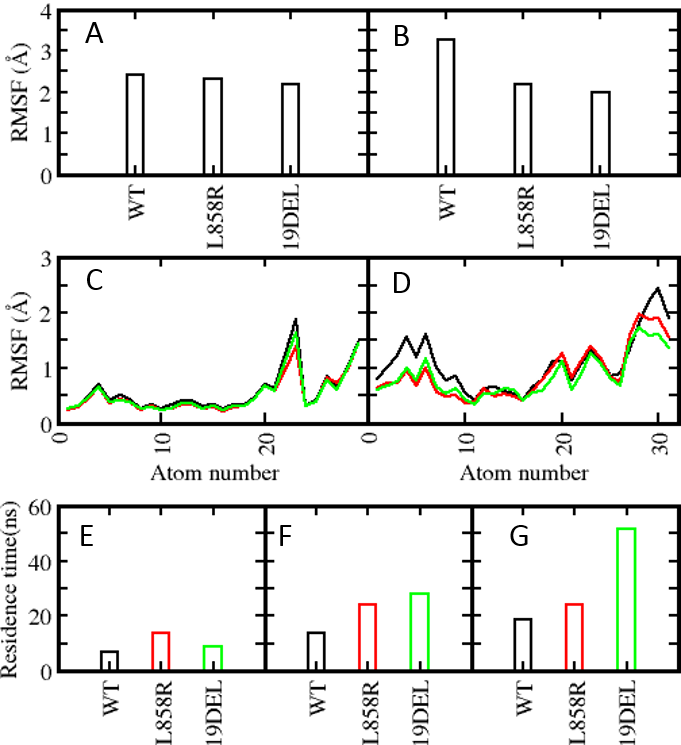


**Figure S6:** Rmsf of conformations of the αC-helix sampled during the MD simulations of *EGFR*^WT^, *EGFR^L858R^, EGFR^19del^* bound with (A) Erlotinib and (B) Gefitinib. Atomic rmsf of the conformations of (C) Erlotinib and (D) Gefitinib bound to *EGFR^WT^* (black) *EGFR^L858R^* (red) *EGFR^19del^* (green) sampled during the MD simulations. (C) Residence times of water molecules observed during the MD simulations of *EGFR^WT^* (black), *EGFR^L858R^* (red), *EGFR^19del^* (green) bound to (E) Erlotinib, (F) Gefitinib and (G) Afatinib.


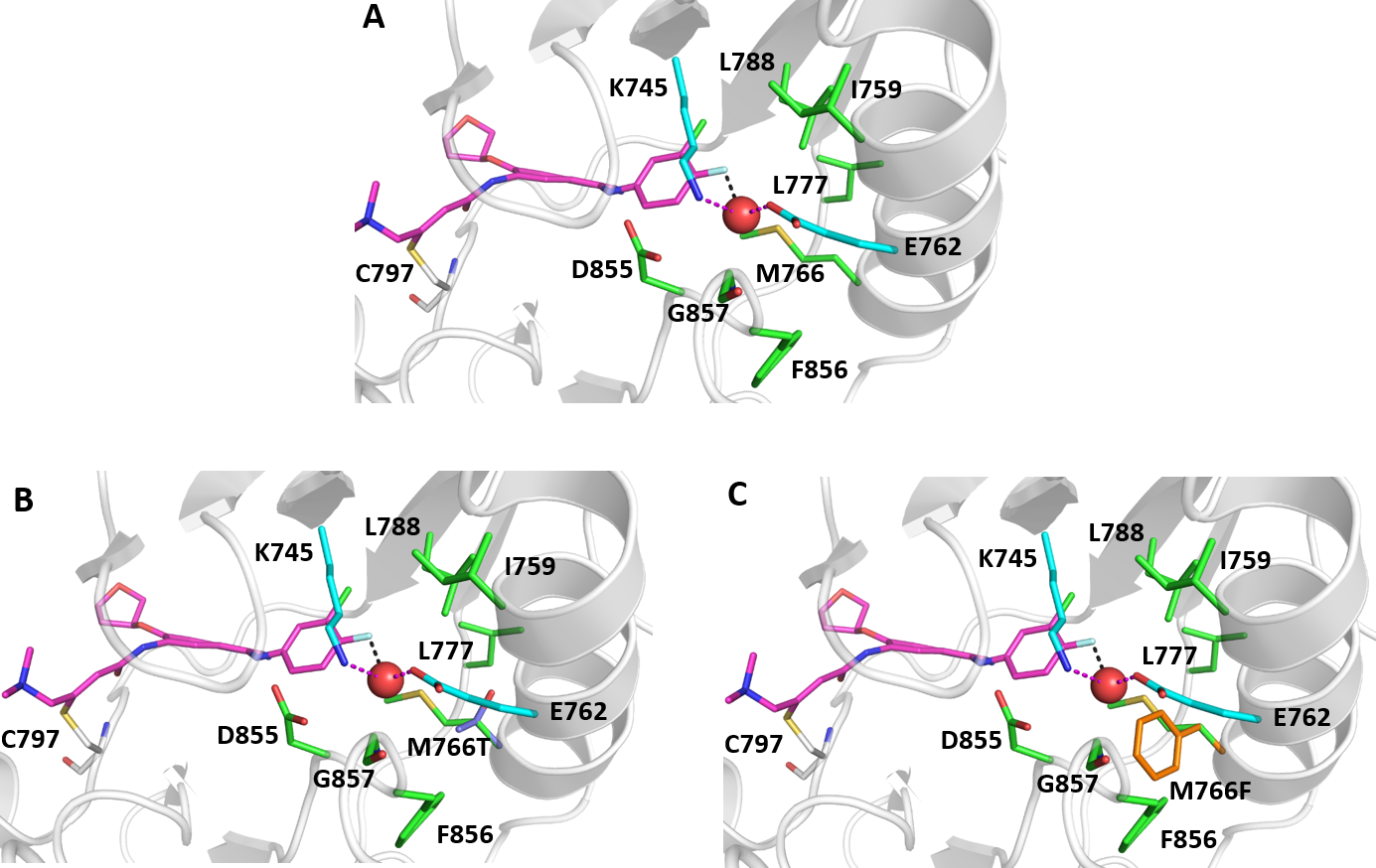


**Figure S7:** Structure of Afatinib (magenta) bound to *EGFR^19del^* (grey) sampled during the MD simulations. A single water molecule (red sphere) was observed to mediate interactions between the halogen of Afatinib and the αC helix of *EGFR^19del^*. Hydrogen bond (magenta) and halogen bond (black) interactions are shown as dotted lines. The critical residues that make the salt bridge (K745 and E762) are shown in cyan sticks. All SNPs within 5 Å of the water molecule are shown as green sticks in (A) and are L777Q, M766T/F, I759V/N, L788F, D855N/G, F856S/L/Y and G857V/R/E. To speculate on the likely effects of the SNPS on the water molecule and hence the interactions with Afatinib, two SNPs have been modelled without any optimization of the resulting changes: M766T in (B) and M766F in (C). It is clear that the resulting changes in the shape of the M766 sidechain will affect the water position, perhaps the number of waters and the interactions of Afatinib and hence its affinity.

**Supplementary Video 1:** MD simulation showing that the R858 in *EGFR^L858R^* flips out and interacts with the negatively charged residue E758.
